# The Use of Mixed Generalized Additive Modeling to Assess the Effect of Temperature on the Usage of Emergency Electrocardiography Examination among the Elderly in Shanghai

**DOI:** 10.1371/journal.pone.0100284

**Published:** 2014-06-17

**Authors:** Wei-ping Ma, Shuo Gu, Yi Wang, Xian-jing Zhang, Ai-rong Wang, Nai-qing Zhao, Yan-yan Song

**Affiliations:** 1 Department of Biostatistics and Social Medicine, School of Public Health, Fudan University, Shanghai, China; 2 Shanghai Children’s Medical Center, Affiliated to Shanghai Jiaotong University School of Medicine, Shanghai, China; 3 Children’s Hospital of Fudan University, Shanghai, China; 4 Shang Hai Medical Insurance Administration, Shanghai, China; 5 Department of Biostatistics, School of Medicine, Shanghai Jiaotong University, Shanghai, China; The Ohio State University, United States of America

## Abstract

**Background:**

Acute coronary artery diseases have been observed to be associated with some meteorological variables. But few of the previous studies considered autocorrelated outcomes. Electrocardiography is a widely used tool in the initial diagnosis of acute cardiovascular events, and emergency electrocardiography counts were shown to be highly correlated with acute myocardial infarction in our pilot study, hence a good index of prediction for acute cardiovascular events morbidity among the elderly. To indirectly assess the impact of temperature on the number of acute cardiovascular events, we studied the association between temperature and emergency electrocardiography counts while considering autocorrelated nature of the response variables.

**Methods:**

We collected daily emergency electrocardiography counts for elderly females and males in Shanghai from 2007 to middle 2012, and studied temperature and other effects on these data using Mixed Generalized Additive Modelling methods. Delayed temperature effect distribution was described as the weighted average of the temperatures within 3 days before the counts was recorded. Autoregressive random effects were used in the model to describe the autocorrelation of the response variables.

**Main Results:**

Temperature effect was observed to be piecewise linearly associated with the logarithm of emergency electrocardiography counts. The optimal weights of the delayed temperature effect distribution were obtained from the model estimation. The weights of lag-1 were the maximums, significantly greater than the weights of lag-2 and lag-3 for both females and males. The model showed good fit with R^2^ values of 0.860 for females and 0.856 for males.

**Conclusion:**

From the mixed generalized additive model, we infer that during cold and mild days, the number of emergency electrocardiography counts increase as temperature effect decreases, while during hot days, counts increase as temperature effect increases. Similar properties could be inferred for the occurrence of cardiovascular events.

## Background

Morbidity or mortality of cardiovascular diseases has been widely studied throughout the world. Seasonal variations has been observed in the historical observations of coronary heart disease such as myocardial infarction (MI), and the seasonality might due to the change of weather. [1], [2], [3] At temperatures below 12°C, cold extremities and slight lowering of core temperature can induce short term increases in blood pressure. Raised blood pressure and increased blood viscosity in moderate cold may be important causal factors in increasing winter morbidity and mortality due to heart attacks and strokes [4].

Both seasonal effects and certain climatic variables have been found to be directly associated with cardiovascular disease morbidity or mortality in previous studies. Denet et al. (1999) studied the effect of atmospheric temperature and pressure on the occurrence of MI and coronary deaths using a Poisson linear regression model, and found out that a 10°C decrease of atmospheric temperature was associated with a 13% increase in event rates. [5] However the linear assumption might be too stringent in representing the real association. Some studies included seasonal effects such as months [6] and seasons [7] to explain some of the nonlinear pattern in the association, but this might affect the estimation of the temperature effect since temperature has also a strong seasonal trend.

Ren et al. (2006) studied the environment effects on both cardiovascular hospital admissions and cardiovascular mortality. They built Poisson generalized additive models on the health outcomes using nonparametric regression function of the temperature and PM10 as the main effects, together with regression splines of the season factor and rainfall as confounding effects. They observed some nonlinear association between temperature and the health outcomes through the model fitting. [8] But all these studies did not consider the autocorrelation of the dependent variable. Michelozzi et al. (2008) specified a first-order autoregressive structure of the daily hospital admissions for respiratory and cardiovascular causes, [9] implying that the independent error assumption might lead to biased estimation with the support of some simulation studies [10].

A temperature-mortality relationship has also been observed, especially among the elderly, in Chinese populations. A nonlinear trend was observed in the association between temperature and mortality from coronary artery disease and cerebral infarction. In the elderly, the risk of cerebral infarction reached its minimum at 27–29°C and the risk of coronary artery disease reached a minimum at 26–29°C. The risks both increased as the temperature reduced from the minimum regions in an approximately exponential manner (linear risk ratio) and went higher as the temperature increased from the minimum regions. [11] But the association between temperature and disease mortality might be different from the association between temperature and disease prevalence.

In the clinical assessment of chest pain, electrocardiography (ECG) is an essential adjunct to clinical history and physical examination. It is a widely used tool in the first step of diagnosing coronary artery diseases such as acute MI (AMI). [12] ECG examination is often firstly performed for elderly emergency patients with acute cardiovascular event symptoms, and we empirically treat these people as patients with suspected cardiovascular events.

To illustrate the association between emergency ECG counts and acute cardiovascular event counts, we designed a pilot study. Since the number of acute cardiovascular event were not recorded in the official health data system, we use the AMI data as a representation. We collected ECG and AMI data for elderly people in Shanghai in July 2011 and December 2011. And we fitted a linear regression model on the number of AMI cases using emergency ECG counts and Gender as predictors separately on the data of the two months, in order to observe how the MI and ECG counts correlated under different temperature conditions.

From those aspects above, we choose to investigate the impact of temperature variations on emergency ECG counts in our study.

As we described, most of the previous studies applied Poisson regression and generalized additive models (GAM) with independent Poisson distributed errors to estimate the weather effects on cardiovascular disease events. However, this may not capture the autocorrelation of the response variable, and can lead to biased estimation of the association.

To reduce the estimation bias we considered the dependent variable to be autocorrelated and fitted mixed generalized additive model (MGAM) [10] on the emergency ECG counts using natural splines of delayed temperature effects, while controlling for time trends and day-of-the-week (DOW) as confounding variables.

Some studies investigated the significance of short term delayed effects of temperature on MI cases. [13] Delayed effects within 3 days has been observed significantly. [8], [14]–[17] Here we also assumed that the temperature effects on ECG counts to be delayed within 3 days.

Therefore in this study, we accessed the association between temperature effects and the morbidity of acute cardiovascular event such as acute myocardial infarction (AMI) among elderly Chinese by separately fitting the MGAM model on the ECG data of both elderly females and males in Shanghai recorded from early 2007 to middle 2012.

## Materials and Methods

### Ethics Statement

Only total daily counts of the number of emergency ECG examination were collected. The study did not involve individual participants, and no individual patient information was collected.

### Data Description and Pilot Study

Shanghai is the most populous city located in the eastern part of China with a mild subtropical climate and abundant rainfall. It has a total area of 6,341 km^2^ with a total resident population of 14.2 million at the end of 2011.

The Emergency ECG data for the elderly (age over 65 years) were from the Official Medicare Database in Shanghai. The daily meteorological data (including minimum, maximum, and mean temperature) were from the Shanghai Meteorological Bureau. The data were measured at a fixed-site station located in the Xuhui District of Shanghai.

In the pilot study we also collected the daily number of AMI cases for elderly people in Shanghai in December 2011. We fitted a linear regression model on the AMI counts using emergency ECG counts and Gender as predictors, to explore the association between emergency ECG counts and AMI counts.

The ECG data, AMI data and meteorological data were validated by an independent auditing team.

### Statistical Analysis

We fitted GAM and MGAM [18] with natural splines of the covariates using a logarithm link function for ECG counts. Temperature, DOW and some other time-variant covariates were considered as fixed effects, while the autoregressive error term was treated as a random effect.

The GAM model was expressed as
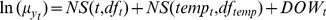
(I)


Here the conditional distribution of ECG counts at the *t*th day approximately followed a Poisson distribution with expectation 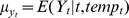. 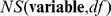 was a natural spline function with degree of freedom 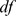, and the lagged temperature effect 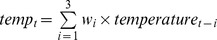 was the weighted average of the temperatures within 3 days before the day 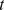, with 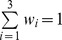. 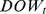 was a dummy variable for DOW effect. We also introduced a natural spline function 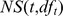 to estimate the time trend representing the time-variant effects on ECG counts.

If we consider the ECG counts as a typical time series, then the autocorrelation pattern can be expressed in (II)–(IV)
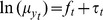
(II)

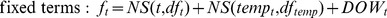
(III)

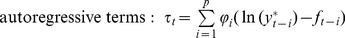
(IV)


Here 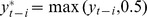, 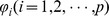 was the coefficient of the autoregressive random effect. When 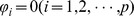, expressions (II)–(IV) would reduce to (I), the GAM.

We used the GAM model to determine the degree of freedom of the natural spline function for time trend 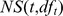 because the random effect was a zero-mean error term. Model (I) can be rewritten as
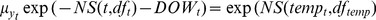
(V)


The temperature effect on the right side has a strong and stable seasonality trend, and the same for the left side. Thus we selected a suitable degree of freedom to make the seasonality of 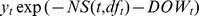 invariant through different years. The estimation of the temperature effect could then be less biased by eliminating the time trend. The seasonality was illustrated in the scatter plot of 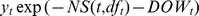 against time 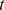, and the selection of 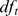 should be as small as possible to avoid underestimating the temperature effect.

Moreover the spline function of 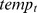 should be sufficiently smooth. Therefore we preselected several suitable degrees of freedom for the temperature effect, and then chose the optimal one according to the Akaike Information Criterion (AIC). We selected the order of the autoregressive error term p on the condition that the partial autocorrelation and autocorrelation could both fall between [−0.1, 0.1], and the optimal value was also decided by the AIC.

The parameters (including the weights 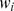
s) in both GAM and MGAM were estimated by maximum partial likelihood method using Newton’s method. All of the analysis was conducted in the statistical software R (version 2.14). The GAM estimation was performed using the R package ‘GAM’, [19] and the MGAM estimation was done using the R function ‘gamwithAR’ [10].

## Results

### Data Overview

The elderly population in Shanghai was about 2.1 million in 2007 (0.95 million males and 1.16 million females) and was relatively stable during the whole study period (2007–2012).

**Figure 1:**
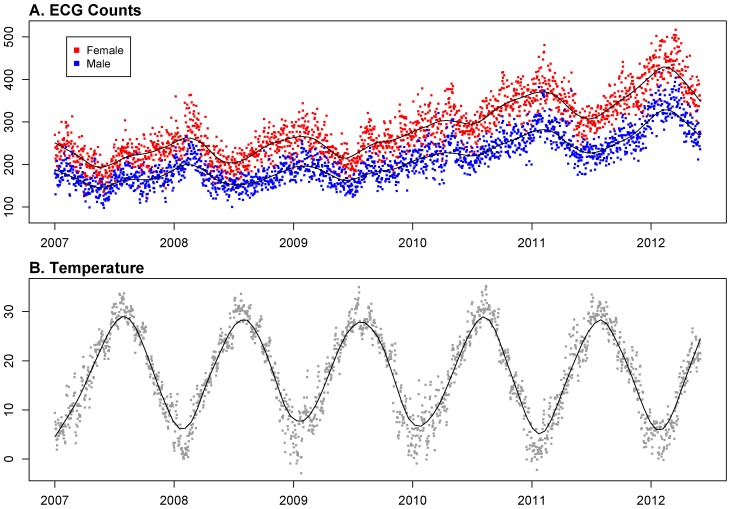
Scatter plot s of ECG counts and Temperature v. s. Time. A. Daily emergency ECG counts in female (red dots) and male (blue dots) elderly group from 2007 to middle 2012, with the solid lines: Lowess estimation of ECG counts. B. Daily averaged temperature from 2007 to middle 2012, with the solid line: Lowess estimation of averaged temperature.

There were a total of 981,974 ECG counts from 418,960 males and 563,014 females between early 2007 and middle 2012 (1,978 days), and the daily average was 284.64 for female and 211.81 for male. More details are provided in Tables 1–2 and Figure 1A. During 2007 to 2012, the range of the 24 h mean temperature was from −2.9°C to 35.2°C (Figure 1B).

**Table 1:** Summary of Daily Emergency ECG Counts of Elderly Female in Shanghai.

Year	Mean	SD	Q1	median	Q3	Min	Max
2007	219.52	33.22	197	221	241	122	313
2008	238.20	40.38	208	235	264	143	364
2009	247.73	36.67	221	248	273	160	344
2010	314.90	41.23	284	314	342	209	417
2011	349.06	44.71	317	347	378	238	481
2012	414.52	49.81	375	417	451	277	517

**Table 2:** Summary of Daily Emergency ECG Counts of Elderly Male in Shanghai.

Year	Mean	SD	Q1	median	Q3	Min	Max
2007	162.88	24.62	147	164	179	98	241
2008	175.09	29.62	154	170.5	193.75	100	266
2009	183.61	26.63	164	183	203	124	255
2010	234.10	30.52	212	232	254	154	321
2011	259.48	38.33	233	254	284	176	382
2012	317.86	39.42	292	320	344.5	212	417

### Pilot Study and Exploratory Analysis

**Figure 2:**
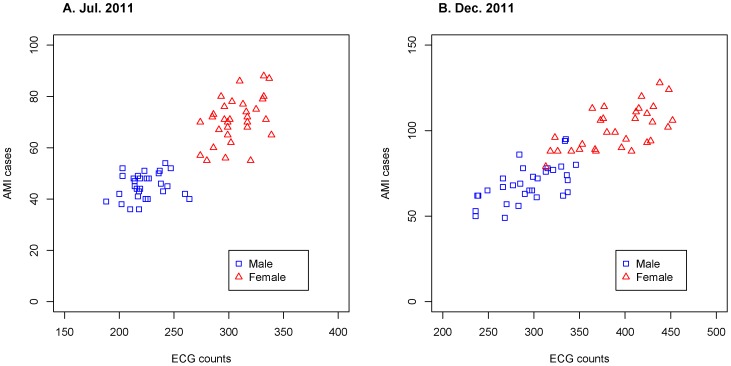
Scatter plot s of MI cases numbers v. s. ECG counts. A. Scatter plot of daily MI cases numbers v. s. daily emergency ECG counts in female (red triangle) and male (blue square) elderly group in the month of July 2011. B. Scatter plot of daily MI cases numbers v. s. daily emergency ECG counts in female (red triangle) and male (blue square) elderly group in the month of December 2011.

In the pilot study, the data of AMI counts and emergency ECG counts were showing a strong linear association (Figure 2). The left part of the figure demonstrated the data of AMI counts and ECG counts in July 2011, and the right part showed the data in December 2011. The results of linear regression model also showed that number of predicted AMI counts were highly correlated with the actual AMI counts (correlation coefficient being 0.8991 for data in July and 0.8877 for data in December).

**Figure 3:**
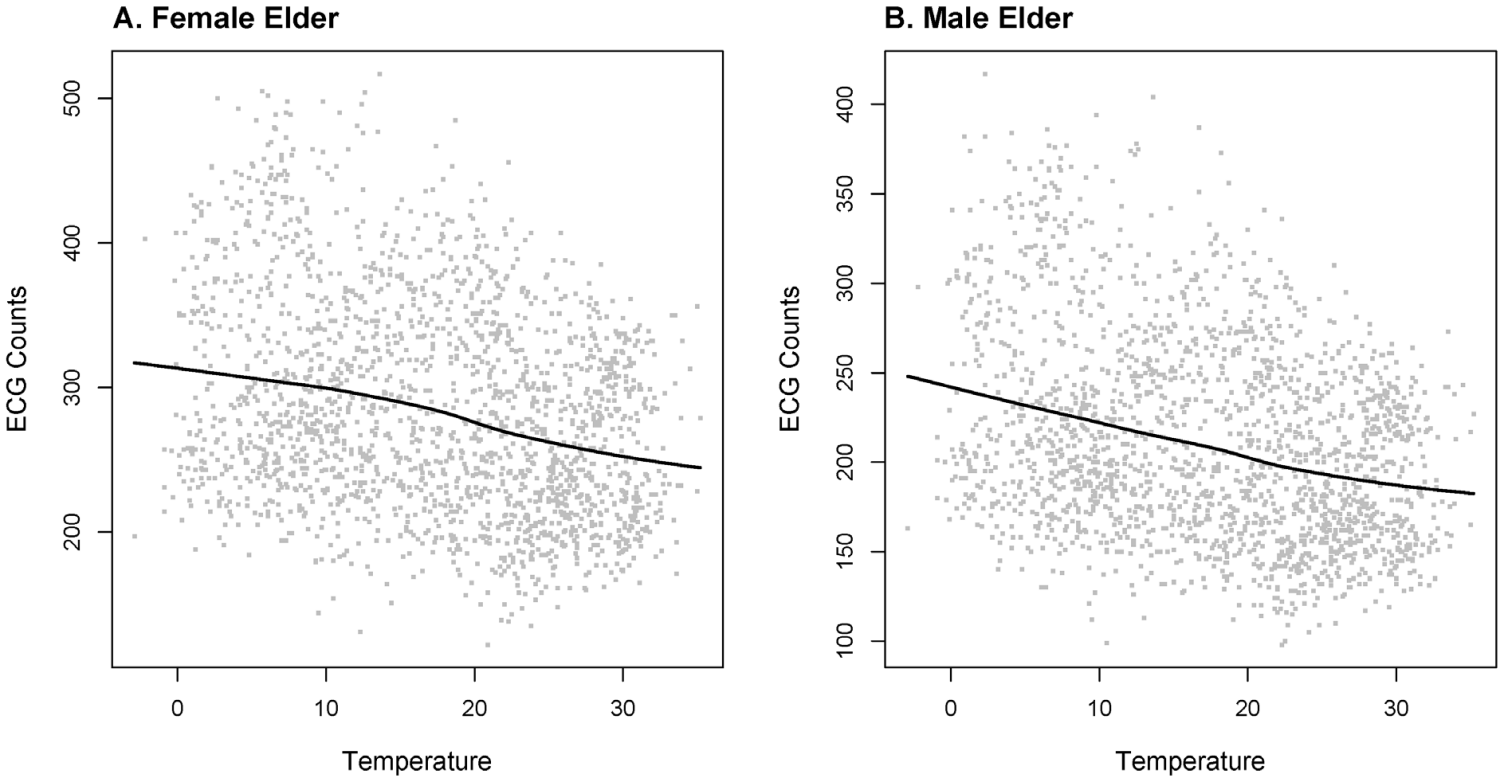
Scatter plot of BD counts v. s. Temperature from 2007 to middle 2012. A. Scatter plot of BD counts v. s. Temperature in female elderly group, with the solid black line: Lowess estimator of the ECG counts against daily average temperature. B. Scatter plot of BD counts v. s. Temperature in male elderly group, with the solid black line: Lowess estimator of the ECG counts against daily average temperature.

From Figure 3, we can roughly observe negative statistical associations between temperature and female ECG counts (Spearman correlation coefficient −0.29) and between temperature and male ECG counts (Spearman correlation coefficient −0.31).

### Selection of Degree of Freedom and Autoregressive Order

**Figure 4:**
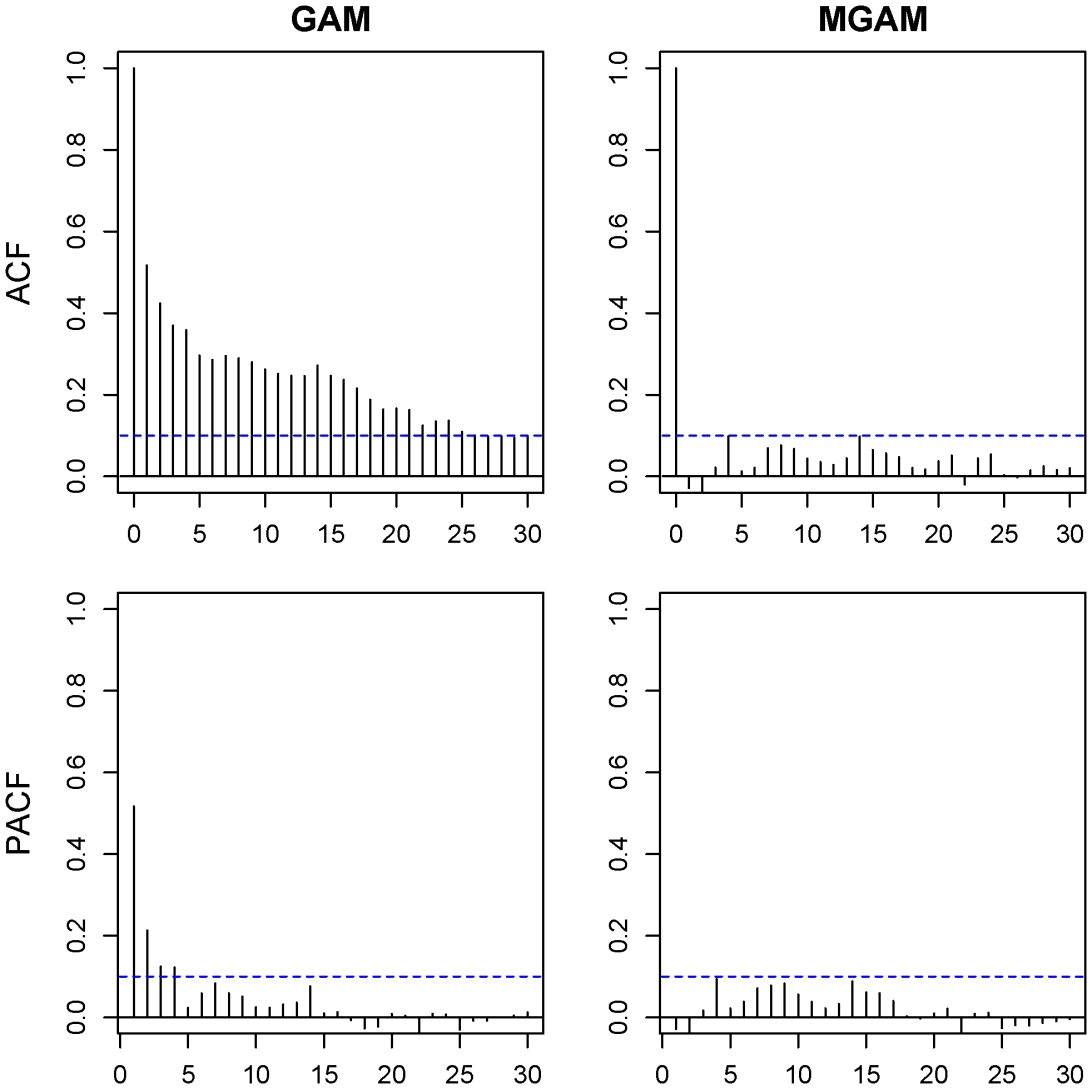
Residual autocorrelation and partial autocorrelation of GAM and MGAM in female elderly group. Upper left is the autocorrelation function (ACF) of GAM residuals. Upper right is the ACF of MGAM residuals. Lower left is the partial autocorrelation function (PACF) of GAM residuals. Lower right is the PACF of MGAM residuals.

Before we fit the MGAM model, we selected proper degree of freedom and the order of autoregressive error term. For female ECG counts, we selected 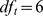 and 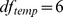 using the methods described in the previous section. However the ACF and PACF plots of the residuals showed that the residual autocorrelation of GAM, 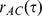 exceeded the uncorrelated bound 0.10 for some nonzero lag 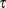 and the partial autocorrelation 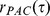 also exceeded the bound for some lag 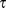 (Figure 4). Then we use the same degrees of freedom (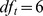 and 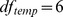) to fit the MGAM and the ACF and PACF plots did not show obvious autocorrelation: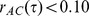 for all nonzero lags 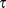 and 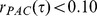 for all lags 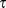 (Figure 4) when the autocorrelation order 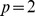. The model showed good fit under the selected degrees of freedom for the female ECG data with a R^2^ value of 0.860 under MGAM.

**Figure 5:**
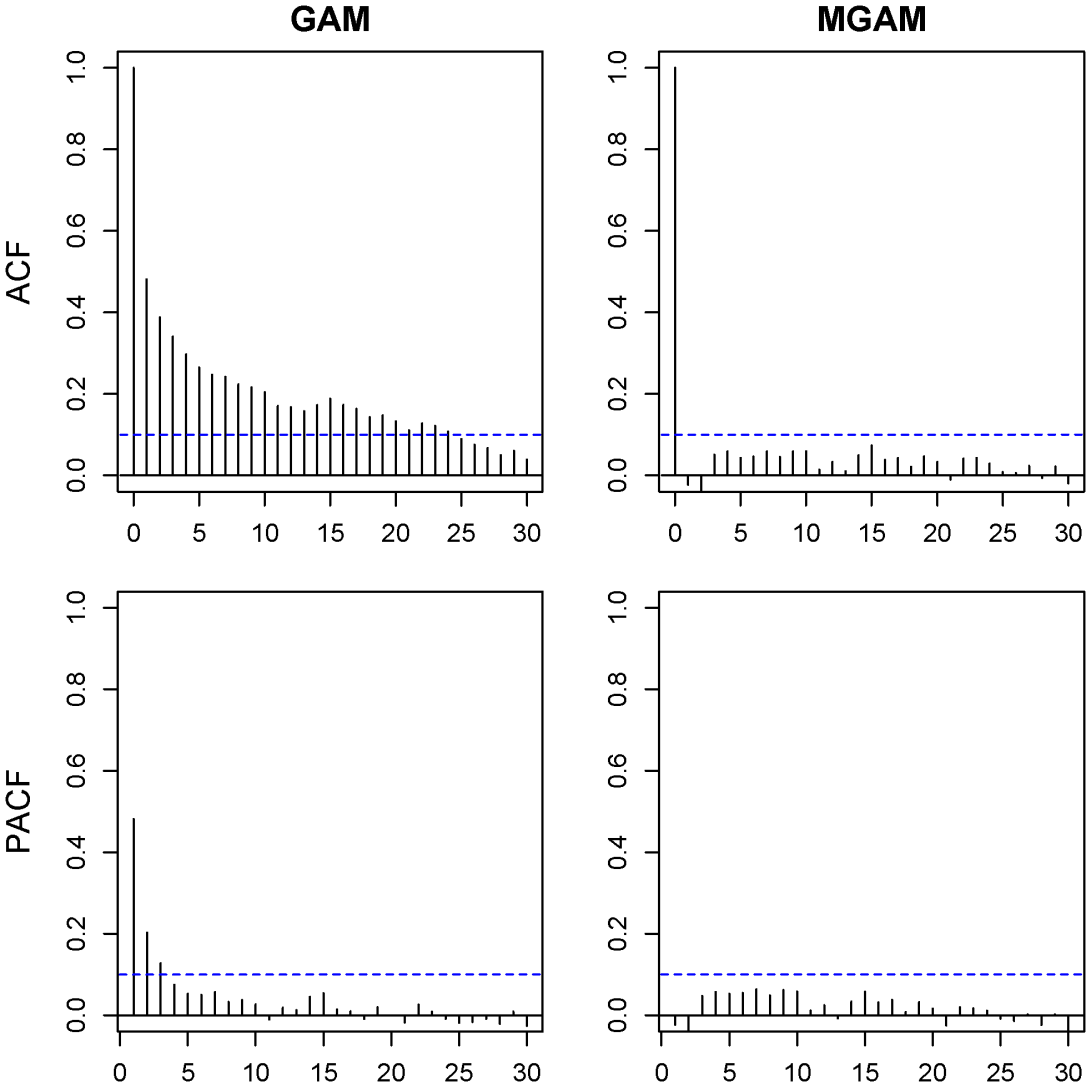
Residual autocorrelation and partial autocorrelation of GAM and MGAM in male elderly group. Upper left is the autocorrelation function (ACF) of GAM residuals. Upper right is the ACF of MGAM residuals. Lower left is the partial autocorrelation function (PACF) of GAM residuals. Lower right is the PACF of MGAM residuals.

Similar results could be obtained from the male ECG data. The degrees of freedom in the spline function were selected as 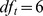 and 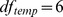, and the order of the autocorrelation was 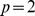 (Figure 5). The model also showed a good fit with a R^2^ value of 0.856.

### Results of MGAM Estimation

The estimation of the temperature effects for both GAM and Mixed GAM were adjusted by the time trends and the DOW effects.

**Figure 6:**
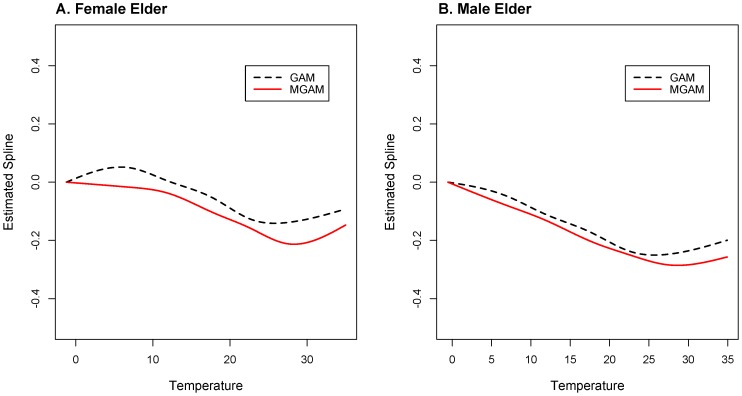
Estimated spline for temperature effect of MGAM and GAM in both data sets. A. Temperature effect of MGAM and GAM estimated from female elderly group data. B. Temperature effect of MGAM and GAM estimated from male elderly group data. The red solid lines in both plots are the estimated spline for temperature effects on ECG counts from MGAM model, and the black dashed lines are the estimated spline for temperature effects on ECG counts from GAM model.

The temperature effect curves were displayed in Figure 6. The temperature effects in the left panel were estimated from female ECG counts, while the right panel was from male ECG counts. For the female ECG counts (Figure 6A), we can see that the temperature curve of the Mixed GAM was V-shaped with the minimums occurring at 27–30°C. The curve went down from 0°C to 27°C, and rose up from 30°C to 35°C. The decreasing part followed a piecewise linear trend with the slope being −0.0024 from 0°C to 11°C (corresponding Risk Ratio (RR) for a 1°C change in temperature was 0.998 and 0.988 for a 5°C change) and −0.0116 from 11°C to 27°C (corresponding RR 0.988 for a 1°C change and 0.944 for a 5°C change). The increasing part also followed a linear trend with the slope being 0.0113 from 30°C to 35°C (corresponding RR 1.011 for a 1°C change or 1.058 for a 5°C change).

From the MGAM estimation of temperature effect on male ECG counts (Figure 6B), we found a similar V-shaped trend with the minimum at 27–30°C. The effect decreased linearly from 0°C to 27°C with slope −0.0107 (corresponding RR was 0.989 for a 1°C change or 0.948 for a 5°C change) and increased linearly from 30°C to 35°C with slope 0.0050 (corresponding RR was 1.005 for a 1°C change or 1.025 for a 5°C change).

The GAM results were different from the MGAM results. For female ECG counts, the temperature trend increased from 0°C to 7°C, decreased in the moderate temperature region (from 7°C to 22°C), reached a minimum at 22–24°C, and increased again above 24°C. While for male ECG counts, the temperature trend was V-shaped with the minimum at 24–26°C.

The temperature effect in model (I) was the weighted average of temperatures from the day of lag 1 to the day of lag 3 of the ECG count time 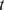. The optimal weights estimated for female ECG counts were 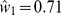,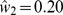,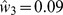. The weight of lag-1 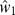 was the maximum of the three, and the other two were close. The difference between 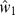 and the other two weights was statistically significant with p-value 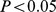. 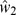, and 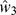 were not significantly different from each other. Therefore we could assign equal weights 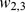 to them, and the estimator 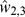 was 0.14.

The optimal weights estimated from male ECG counts were 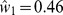,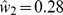,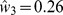. The weight of lag-1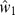 was also the maximum of the three, and the other two were close. The difference between 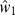 and the other two weights was slightly significant at 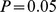. 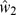 and 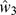 were not significantly different from each other. Therefore we could also assign equal weights 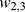 to them, and the estimator 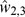 was 0.27.

**Figure 7:**
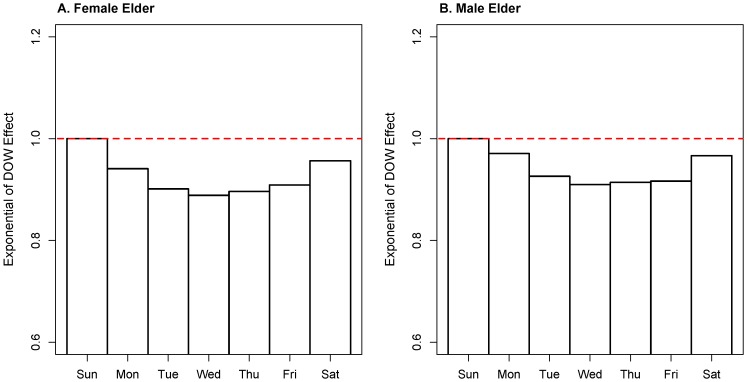
Exponential of DOW effect estimated by MGAM. A. Exponential of DOW effect from female elderly group. B. Exponential of DOW effect from male elderly group.

The DOW effect was similar for both females (Figure 7A) and males (Figure 7B). The maximum effects occurred on Sunday, and the effects showed a U-shaped trend within a week with the minimums occurring on Wednesday.

## Discussion

ECG examination is generally required for emergency patients suffering from heart discomfort. In the elderly population (age over 65 years), patients requiring ECG monitoring are likely to have acute cardiovascular events including AMI. As validated in the pilot study, the ECG counts were correlated with AMI counts on both hot and cold weather, we could view the emergency ECG counts as an index representing for the number of acute cardiovascular events. Moreover the impact of temperature on ECG counts could be an indirect evidence of the impact of temperature on AMI event numbers.

The ECG count data could be viewed as a typical time series, and may have strong autocorrelation. To capture the autocorrelation we fitted the data with MGAM which was proposed by Yang et al. (2012) as a modification of GAM to study the daily temperature effect on mortality in Shanghai from 2001 to 2004. Simulation results showed MGAM provides better estimates than GAM when there is autocorrelation in the dependent variable, and the autocorrelation function (ACF) and partial autocorrelation function (PACF) of the Pearson residuals also showed better fit [10].

Similarly in our study, when selecting the degree of freedoms, we fitted the data with both GAM and MGAM, and the residuals shows different properties. The ACF and PACF plots of GAM residuals showed obvious autocorrelation, which implied that the results of the GAM contradicted its assumption due to the autocorrelation of the data. On the other side the plots of MGAM residuals showed very week autocorrelations. Those results implied that MGAM may fit the data better than GAM.

Intuitively, there could be some short term delay between extreme weather conditions and the occurrence of acute cardiovascular diseases. This is supported in many previous research; likewise, the impact of temperature on ECG count would also be delayed. Some of the previous research studied the delayed effects separately using different time lags. [8], [16] For example, Lin et al. (2009) reported that the greatest number of hospital admissions for cardiovascular diseases was 1–3 days after an increase in temperature, and the increased risks were 2.5%, 2.1% and 3.6% at 1, 2, and 3 days later, respectively, for each °C increase in apparent temperature above the threshold of 29.4°C; [16] And thus could not specify the combined effect of those time lags. And some of them used the average of the delayed time periods as the real temperature effect. [17], [20] But this approach could not tell the difference between different lags. Some studies put different lagged temperatures as different covariates into one model and estimate all the linear and quadratic coefficients simultaneously. [14] This may provide more information about the delayed temperature distribution, but could not estimate the association efficiently especially when the model needs to be more precise.

In our study, we also assumed the impact of temperature on the ECG counts was delayed for a few days, but other than using the temperature in a particular lag or take average of the temperature in different lags, we computed the weighted mean of the lagged daily averaged temperature within 3 days before the ECG count, and optimally selected the weights by the data. This would be a good way to properly capture the distribution of the delayed temperature effect in a time period.

The optimal weights estimated from female ECG counts were 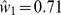,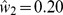, 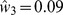, and the weight of the one day lagged was significantly larger than the weights of the other lags. This implied a stronger impact of the one day lagged temperature on female ECG counts. The optimal weights estimated from male ECG counts were 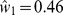, 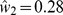, 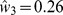. The difference of the weights between the one day lagged and the others was slightly significant (*P = *0.05) which also means a stronger impact of the one day lagged temperature on male ECG counts.

The estimated temperature effect curves for elderly females and males were similar. Both curves went down from 0°C to 27°C, and rose up from 30°C to 35°C. This roughly decreasing tendency coincided with the negative correlations between ECG counts and temperature which were summarized in the exploratory analysis. The increasing part of the curve in the extremely high temperature region could be explained by the decreasing of body tolerance to hot weather among the elderly. Some other researchers studying the impact of temperature on cardiovascular diseases also found out similar trend of the temperature effect. [5], [14], [16] The similarity would also support what we concluded from the pilot study.

The temperature effect curves for females and males behaved similarly under moderate temperatures (11°C to 27°C) with the slope being −0.0116 (female) and −0.0107 (male), which meant that ECG counts would decrease 5.6% (female) and 5.2% (male) for every 5°C escalation in temperature.

There were some differences between the two curves under extreme temperatures (0°C to 11°C, or 30°C to 35°C). The temperature curve showed linear patterns in these temperature regions.

The slope of the temperature effect for females was −0.0024 from 0°C to 11°C (risk decreases 1.2% as the temperature increases 5°C) and the slope for males was −0.0107 (risk decreases 5.2% as the temperature increases 5°C). Thus the risk decreased 4% more for males than for females when temperature was relatively low (0°C to 11°C). This might be due to the improvement of cardiovascular function in males being greater than that for female when temperature goes up in cold weather.

From 30–35°C, the slope of the temperature effect for females was 0.0113 (risk increases 5.8% as the temperature increases 5°C), and the slope for males was 0.0050 (risk decreases 2.5% as the temperature increases 5°C). Thus the risk increased 3.3% more for females than males when temperature was relatively high (30°C to 35°C). This might be because elderly females are generally less tolerant to hot weather than elderly males.

## Conclusion

With the autocorrelated random effects, the MGAM could analyze the association between the daily emergency ECG counts and temperature effect much better than GAM. The delayed temperature distribution was obtained by estimation the optimal weights of temperature in three days before the day of the ECG.

Based on the result of pilot study, additionally validated by some other research, we could infer that the impact of temperature on ECG counts was an indirect evidence of the impact of temperature on AMI event numbers.

From our results we could indirectly obtain: for both elderly females and males, the number of AMI events were positively associated with the temperature as the temperature were extremely high, and negatively associated with the temperature under the cold and moderate weather.
